# Quantitative Trait Loci (QTL) Study Identifies Novel Genomic Regions Associated to Chiari-Like Malformation in Griffon Bruxellois Dogs

**DOI:** 10.1371/journal.pone.0089816

**Published:** 2014-04-16

**Authors:** Philippe Lemay, Susan P. Knowler, Samir Bouasker, Yohann Nédélec, Simon Platt, Courtenay Freeman, Georgina Child, Luis B. Barreiro, Guy A. Rouleau, Clare Rusbridge, Zoha Kibar

**Affiliations:** 1 CHU Sainte Justine Research Center, Université de Montréal, Montreal, Quebec, Canada; 2 Fitzpatrick Referrals, Goldaming, Surrey, United Kingdom; 3 Department of Small Animal Medicine & Surgery, College of Veterinary Medicine, University of Georgia, Athens, Georgia, United States of America; 4 Small Animal Specialist Hospital, Sydney, New South Wales, Australia; 5 Montreal Neurological Institute and McGill University, Montreal, Canada; 6 School of Veterinary Medicine, Faculty of Health & Medical Sciences, University of Surrey, Guildford, Surrey, United Kingdom; University of Queensland, Australia

## Abstract

Chiari-like malformation (CM) is a developmental abnormality of the craniocervical junction that is common in the Griffon Bruxellois (GB) breed with an estimated prevalence of 65%. This disease is characterized by overcrowding of the neural parenchyma at the craniocervical junction and disturbance of cerebrospinal fluid (CSF) flow. The most common clinical sign is pain either as a direct consequence of CM or neuropathic pain as a consequence of secondary syringomyelia. The etiology of CM remains unknown but genetic factors play an important role. To investigate the genetic complexity of the disease, a quantitative trait locus (QTL) approach was adopted. A total of 14 quantitative skull and atlas measurements were taken and were tested for association to CM. Six traits were found to be associated to CM and were subjected to a whole-genome association study using the Illumina canine high density bead chip in 74 GB dogs (50 affected and 24 controls). Linear and mixed regression analyses identified associated single nucleotide polymorphisms (SNPs) on 5 Canis Familiaris Autosomes (CFAs): CFA2, CFA9, CFA12, CFA14 and CFA24. A reconstructed haplotype of 0.53 Mb on CFA2 strongly associated to the height of the cranial fossa (diameter F) and an haplotype of 2.5 Mb on CFA14 associated to both the height of the rostral part of the caudal cranial fossa (AE) and the height of the brain (FG) were significantly associated to CM after 10 000 permutations strengthening their candidacy for this disease (*P = *0.0421, *P* = 0.0094 respectively). The CFA2 QTL harbours the *Sall-1* gene which is an excellent candidate since its orthologue in humans is mutated in Townes-Brocks syndrome which has previously been associated to Chiari malformation I. Our study demonstrates the implication of multiple traits in the etiology of CM and has successfully identified two new QTL associated to CM and a potential candidate gene.

## Introduction

Chiari-like malformation (CM) is a disorder of the craniocervical junction characterized by overcrowding of the neural parenchyma and disturbance of cerebrospinal fluid (CSF) flow [1], [2]. A strong association exists between CM and syringomyelia (SM), a condition where fluid filled cavities (syrinxes or syringes) develop within the central spinal cord and the resulting damage produces clinical signs of pain and variable neurological deficits such as scoliosis and paresis [1], [3]. Disturbance of the normal free flow of CSF through the foramen magnum appears to be a major factor responsible for the formation of a syrinx.

CM is very common in two brachycephalic toy breed dogs, the Cavalier King Charles spaniels (CKCS) and Griffon Bruxellois (GB), with reported frequencies of almost 100% and up to 65% respectively [2], [4], [5]. While the exact cause of the disease remains unknown, a strong association between the brachycephalic skull shape and CM was reported [2], [6]–[8]. The bony changes characteristic for CM are shortening (craniosynostosis) of the basicranium and supraoccipital bone resulting in an insufficient caudal cranial fossa volume. A consistent feature is hindbrain and sometimes forebrain overcrowding with narrowing or obstruction of the CSF channels. The supraoccipital bone indents the cerebellum, which loses its normal rounded shape. In classical CM, the cerebellum and medulla herniate into or through the foramen magnum. However in some individuals the size of cerebellar herniation may be minimal [2]. The craniosynostosis results in compensatory changes by other skull bones including lengthening of the parietal bone and a more horizontally orientated tentorium cerebelli [7], [9]. Correlations between those changes and the brachycephalic features of these breeds suggest that selection for this trait may have been partly responsible for the high prevalence of the disease in those dogs.

CKCS and GB breeds are genetically related. The GB is a brachycephalic toy breed with terrier characteristics, which has origins from the Smousje (an Affenpinscher-like dog), Pug and ruby Toy spaniel [2], [10]. The Toy spaniel is also the main ancestral dog for the CKCS. Pedigree studies as well as the high incidence of CM in these two breeds as compared to other breeds suggest the involvement of genetic factors in its etiology. Incomplete penetrance and variable transmission of CM seem to point toward a complex polygenic mode of inheritance [2], [6], [11], which impedes identification of the predisposing genetic factors using traditional genetic cloning approaches. A more powerful approach to investigate the apparent genetic complexity of CM is quantitative trait loci (QTL) analysis which aims at identifying genes or QTL that determine the disease even though each gene contributes only a small fraction. In fact, QTL analysis has been successfully used in dogs to identify QTLs associated with complex diseases like hip dysplasia or epilepsy demonstrating its power in such genetic investigation studies [12]–[14].

In the present study, we conducted a whole genome QTL association study to identify the genes that predispose to CM in the GB dog breed. Among a total of 14 measurements taken from T1-weighted sagittal Magnetic Resonance Imaging (MRI) of the brain and cranial cervical vertebral column of 92 CM affected GB and 31 unaffected GB, six traits were found to be significantly associated to CM in this breed. A QTL study then performed on those 6 measurements in 50 CM affected GB and 24 unaffected GB identified two loci on Canis Familiaris autosome (CFA) 2 and CFA14 strongly associated to cranial fossa height (Diameter F) and height of the rostral part of the caudal cranial fossa (trait AE). These 2 QTL were then mapped back to CM strengthening their candidacy in the development of the disease.

## Materials and Methods

### Ethics statement

MRI of the brain and cervical region were obtained either for diagnostic reasons or for screening prior to breeding. Blood or saliva was withdrawn at the end of the MRI procedure whilst the dog is still under the effect of the anaesthesia ensuring minimal stress to the animal. In the United Kingdom (UK), Home Office regulations restrict blood sampling for non-diagnostic reasons however if the dog had a blood sample taken for a veterinary diagnostic test and a small amount of excess blood in ethylenediaminetetraacetic acid remained then this sample was submitted to the study. For majority of UK owned dogs, the DNA was collected non-invasively via a sponge that absorbs saliva in the mouth (Oragene-ANIMAL, DNA Genotek, Inc). It was not necessary to obtain approval from an ethical committee as the procedures performed were the necessary diagnostic tests for the animals undergoing veterinary treatment. Consent was obtained from all owners and actual identity of dogs remained anonymous. It is important to note that dogs included in this study were not experimental animals but animals undergoing appropriate veterinary treatment and therefore no additional welfare considerations were required.

### Cohort and phenotypic traits

**Figure 1:**
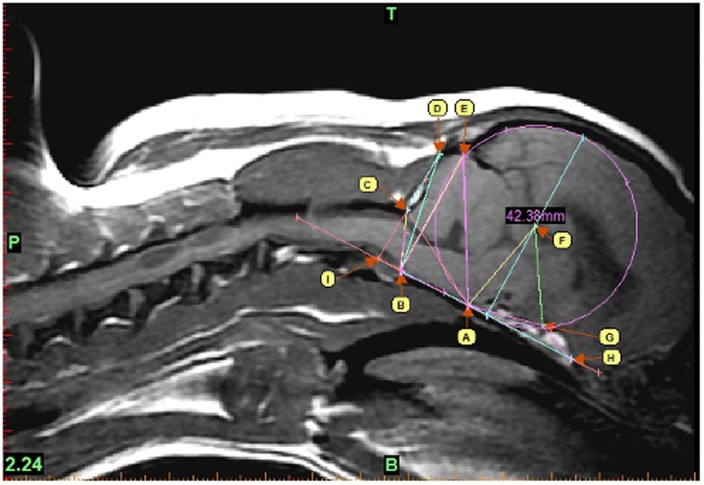
Morphometric measurements of a Griffon Bruxellois skull. Measurements were chosen to maximize coverage of the possible variation associated with CM. All measurements start from one of those 9 points :(A) the dorsum of sphenoid-occipital synchondrosis, (B) the basion of basioccipital bone, (C) the rostral edge of the dorsal lamina of the atlas, (D) the junction between the supraoccipital bone and the occipital crest, (E) the most dorsal point of intersection of the cerebellum with the occipital lobe circle, (F) the center of occipital lobe circle, (G) the optic nerve, (H) the most caudal point of the olfactory bulb and (I) the intersection point with the HA baseline.

A cohort of 154 GB with DNA consisting of 65 males and 89 females with an average age of 4.5±2.6 years was recruited for the quantitative study. DICOM (Digital Imaging and Communications in Medicine) T1-weighted sagittal MRI images of the brain and cervical region of 154 Griffon Bruxellois dogs were analysed in this study. Nine potentially interesting structures were defined: (A) the dorsum of sphenoid-occipital synchondrosis, (B) the basion of basioccipital bone, (C) the rostral edge of the dorsal lamina of the atlas, (D) the junction between the supraoccipital bone and the occipital crest, (E) the most dorsal point of intersection of the cerebellum with the occipital lobe circle, (F) the center of occipital lobe circle and (G) the optic nerve. A cranial baseline passing by A was then defined from (H) the most caudal point of the olfactory bulb to (I) the intersection point with the line passing through the most caudal measurement (CD). The occipital lobe circle was designed by placing it on the cranial baseline and extending it to encompass the occipital lobes (Figure 1). Fourteen measurements were then chosen to maximize the coverage of the morphological changes associated with CM using MIMICS 14.12 Materialise (Technologielaan 15 3001 Leuven Belgium). MIMICS is a software specialized in medical images processing and offer accurate 2D measurements. The obtained measurements were: AB, AE, AH, BC, BD, BE, CD, FG, F-Diameter, angle 1 ABC, angle 2 CAF, angle 3 AID, angle 4 AGH, angle 5 AFG (Figure 1).

The cohort was classified in three categories based on their level of CM affliction as evidenced by MRI. Dogs with round cerebellum shape and no evidence of narrowing or obstruction of the CSF channels were defined as unaffected, dogs with indentation of the cerebellum by the supraoccipital bone and a narrowed but not obstructed CSF channel (signal consistent with CSF between the caudal vermis and the foramen magnum) were defined as mild CM and dogs where the cerebellar vermis is impacted into or herniated through the foramen magnum were defined as affected. Dogs classified as having mild CM showed signs of affliction, but the incomplete penetrance of the phenotype makes it impossible to include them in the affected category. This MRI-based classification resulted in a cohort consisting of 92 CM affected GB, 31 mildly affected GB and 31 CM unaffected GB. Mildly affected dogs were excluded from further phenotypic and genetic studies.

Sex and age were also obtained. Due to absence of normality in certain of those traits, Wilcoxon rank-sum test was used to define the potential implication of those confounding factors in the development of CM. This was done using the sex as a binary trait, while age was clustered in three groups defined as: 0–3 years, 3–5 years, 5+ years.

### Quantitative trait loci analysis

Genotyping data was obtained on 80 dogs with unambiguous phenotypes (excluding mild CM) and sufficient DNA resulting in 53 CM affected GB and 27 CM unaffected GB. DNA was extracted from blood or saliva using the ORAGENE Animal kit (DNAgenotek). Whole genome genotyping was conducted using the Illumina CanineHD Beadchip containing a total of 173 662 SNPs. After filtering for minor allele frequency of 0.1 and SNP genotyping rate of 0.1, 75 399 SNP remained. Subsequent filtering for individual genotyping rate of 0.01 resulted in a cohort of 74 dogs (50 cases and 24 controls). Canfam 2.0 genomic build was used throughout this paper.

Due to certain traits not following normality, association to CM was performed using a Wilcoxon rank sum test in R v3.0.1 [15]. Linear regression analysis was done using Plink V1.07 [16], [17] and correction for stratification was done using GenABEL mixed model approximation routine [18]. The linear model included age as a fixed effect while the mixed model included both age and a kinship matrix as fixed effects to correct for possible stratification problems. Correction for multiple testing was done using Storey's q value method [19] with a q value threshold of 0.05 and was computed using 6×75 399 (6 significant traits tested) informative SNPs. Suggestive of association threshold was fixed at 1×10^−5^ in the linear model. Subsequent haplotype analysis was performed using haploview v4.2 and 10 000 permutations were applied to the identified haplotypes to correct for genomic inflation. Haplotypes positions were inferred from candidate SNPs and their length was increased until a stretch of SNPs with a D′ under 0.5 was encountered suggesting an ancestral recombination. Total variance explained by the traits was defined using Solar v2.0 polygenic function.

Raw and processed data is available for the 80 dogs with genotypes and can be obtained through the GEO website (http://www.ncbi.nlm.nih.gov/geo/) using accession number GSE52221.

## Results

### Quantitative trait association to CM in the GB breed

To capture the morphological variation associated with CM in the GB breed, we identified 14 two dimensional measurements of the skull and the brain on 154 dogs from this breed (figure 1). The dogs were then classified in three categories based on the degree of CM affliction as described in Materials and Methods. Since ambiguous phenotypes can diminish detection power, dogs categorized as mild CM were removed which resulted in a cohort of 123 dogs used for further association studies.

**Figure 2:**
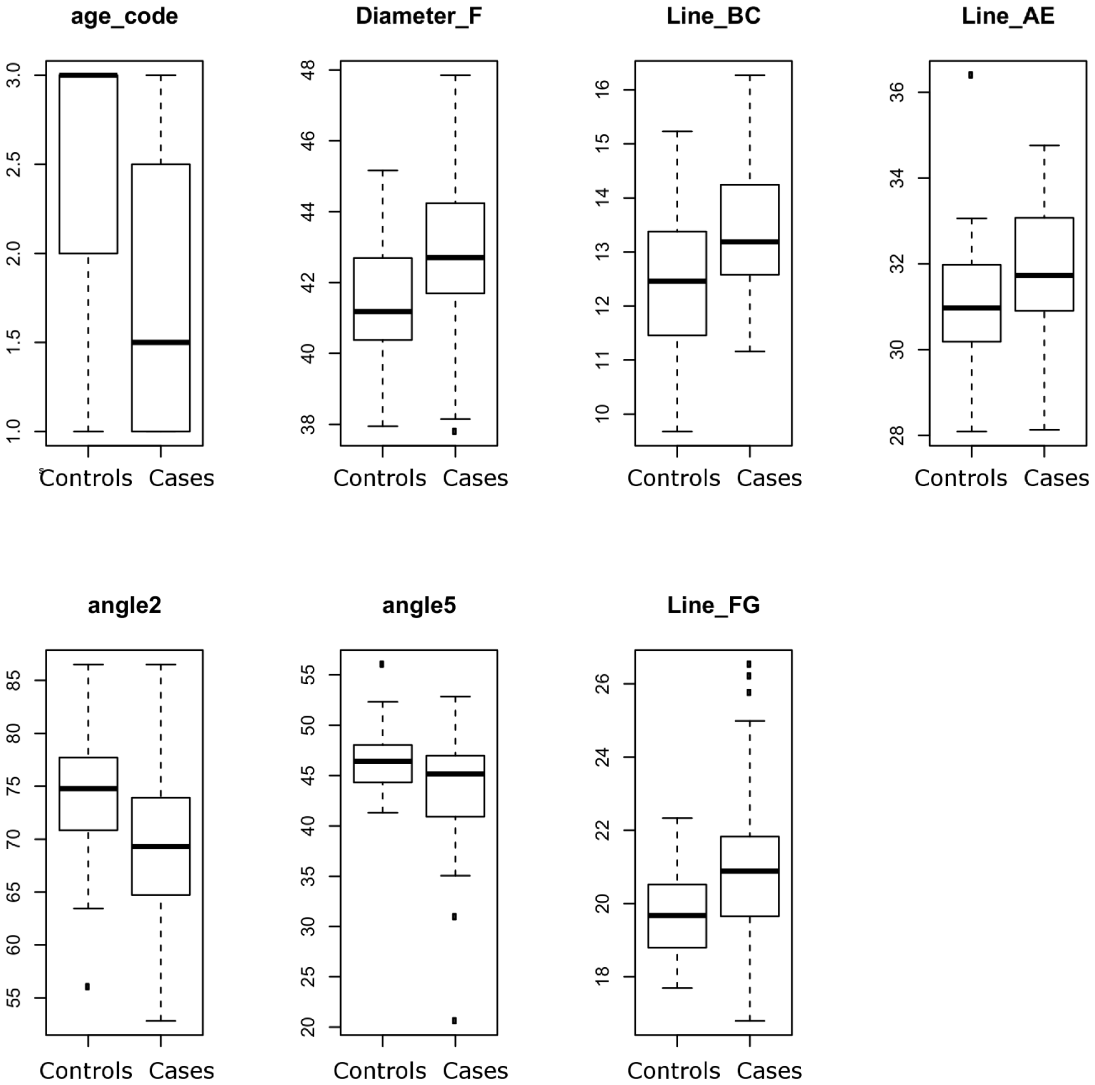
Boxplot of CM significantly associated traits and confounding factors. Boxplot distribution of all 7 CM-significantly associated traits and age code. Boxplots were done on a cohort of 123 GB with unambiguous phenotypes.

Association of the 14 identified quantitative traits to CM in this unambiguous phenotype cohort of 123 dogs was tested using a Wilcoxon rank-sum test and 6 of those passed the significance threshold (*P*<0.05). Diameter F, line BC, line AE, angle 2 CAF, angle 5 AFG, line FG as well as age code were all significantly associated to CM and were therefore further investigated (Table 1 and figure 2). The effect of sex on the development of CM was also tested but did not show any sign of association.

**Table 1:** Quantitative traits that are significantly associated (*P*-value<0.05) to CM following Wilcoxon rank sum test statistic.

Trait	P value
Age code	0.0029
Diameter F	6.2834e-05
Line BC	0.0056
Line AE	0.0200
Angle 2 (CAF)	0.0016
Angle 5 (AFH)	0.02539
Line FG	0.0026

### Whole genome QTL association study of CM in the GB breed using linear and mixed regression models

**Figure 3:**
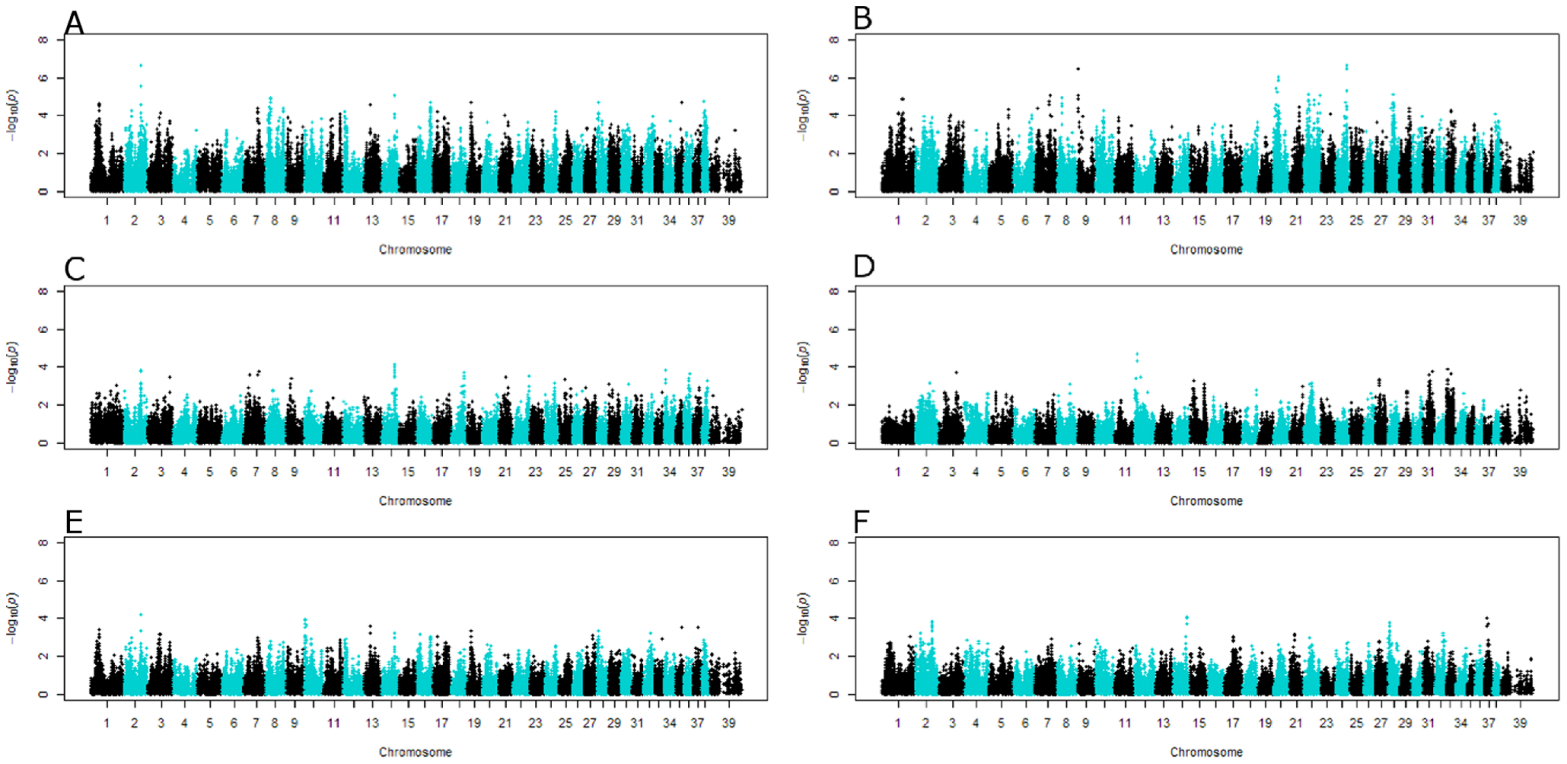
Manhattan plot of the significant QTL obtained by linear or mixed regression models. Manhattan plot of the 2 significant QTL obtained by the linear model in traits length BC (panel A) and Diameter F (panel B) and the 4 traits containing the 3 associated regions in the mixed linear model for traits length AE (panel C), Angle 5 (panel D), Diameter F (panel E) and length FG (panel F).

A total of 80 dogs with sufficient DNA were genotyped and submitted to a whole genome QTL association study. Out of those, 74 dogs had high quality genotyping data and were chosen for further genetic studies. A whole genome association study was conducted for each of the 6 CM-associated quantitative traits in all 74 GB dogs using Plink v1.07 linear regression routine using age as a covariable. This analysis resulted in the identification of one SNP on CFA2 (rs8785068), that was significantly associated to diameter F (*P* = 3.19×10^−7^), 3 SNPs on CFA9 (rs8950347, rs22661153, rs22660327) and 2 SNPs on CFA24 (rs23195408, rs23168064), that were all significantly associated to the line BC (*P* = 1.40×10^−7^, 1.40×10^−7^, 1.40×10^−7^, 8.77×10^−8^ and 1.03×10^−7^ respectively) (table 2 and figure 3).

**Table 2:** QTL significantly associated to Diameter F and Length BC in GB dogs obtained in the linear model and QTL associated to Length AE, Length FG and Angle 5 in the mixed model.

CFA*	Position	SNP	Regression type	Trait	Linear *P* value	Linear *q* value	Mixed *P* value	Mixed *q* value
2	66921327	rs8785068	Linear	Diam F	3.19E-07	0.0203	6.64E-05	0.9612
9	3100030	rs8950347	Linear	BC	1.40E-07	0.0107	1.52E-05	0.9612
9	3167765	rs22661153	Linear	BC	1.40E-07	0.0107	1.52E-05	0.9612
9	3183571	rs22660327	Linear	BC	1.40E-07	0.0107	1.52E-05	0.9612
12	12650887	rs22186794	Mixed	Angle 5	7.67E-05	0.1317	1.89E-05	0.9612
12	12672798	rs24735719	Mixed	Angle 5	0,00015	0.1431	4.53E-05	0.9612
12	12681765	rs8936205	Mixed	Angle 5	0,00015	0.1431	4.53E-05	0.9612
12	12685337	rs8757898	Mixed	Angle 5	0,00015	0.1431	4.53E-05	0.9612
14	51991792	rs22301389	Mixed	AE	5.44E-06	0.0913	6.84E-05	0.9612
14	51700039	rs22292669	Mixed	AE	4.54E-06	0.0913	9.78E-05	0.9612
14	51704846	rs22360350	Mixed	AE	4.54E-06	0.0913	9.78E-05	0.9612
14	52387787	rs22336002	Mixed	AE	7.78E-06	0.1011	9.78E-05	0.9612
14	53687526	rs22284682	Mixed	FG	1.04E-05	0.1067	7.78E-05	0.9612
14	53758809	rs22374739	Mixed	FG	1.82E-05	0.1067	9.34E-05	0.9612
24	43273701	rs23195408	Linear	BC	8.77E-08	0.0107	0.001397	0.9612
24	43276820	rs23168064	Linear	BC	1.03E-07	0.0107	0.001633	0.9612

*CFA = Canis familiaris autosome.

Subsequently, GenABEL mixed model association was used to assess the impact of the dogs' familial affiliations. Age was once more included as a fixed effect in the model. Relatedness bias was also included as a kinship matrix which was computed and included in the model as a fixed effect. With this kind of analysis, we did not detect any statistically significant association to any of the 6 traits analyzed. However, careful analysis of the highest *P* values obtained for each trait led to the identification of a cluster of 4 SNPs on CFA12 and a cluster of 6 SNPs on CFA14 that were associated to angle 5 (CFA12) and to length AE and FG (CFA14). The 4 SNPs on CFA12 were rs22186794, rs24735719, rs8936205, rs8757898 (*P = *1.89×10^−5^, 4.53×10^−5^, 4.53×10^−5^, 4.53×10^−5^ respectively), and those on CFA14 were (rs22301389, rs22292669, rs22360350, rs22336002, rs22284682, rs22374739) (*P = *6.84×10^−5^, 9.78×10^−5^, 9.78×10^−5^, 9.78×10-5, 7.78×10^−5^, 9.34×10^−5^). Notably, the significant SNP on CFA2 (rs8785068) associated to diameter F in the linear model (*P* = 3.19×10^−7^) was also interesting in the mixed model (*P* = 6.64×10^−5^) (table 2 and figure 3).

### Haplotype analysis of the significantly associated QTL

To better assess the association of the 2 SNP clusters identified in the mixed linear model, a multi-marker approach seemed in order. Therefore, QTLs identified in both the linear and the mixed models were subjected to further validation by haplotype association to the CM phenotype. Haplotype reconstruction was done using Haploview v4.2. We identified 5 haplotypes surrounding the QTLs on CFAs 2, 9, 12, 14, 24 that were subsequently tested for association with the CM phenotype. While reconstructed haplotypes in QTL regions on CFA24 did not show any association to CM, significant scores were found for haplotypes on QTL regions of CFA2, CFA9, CFA12 and CFA14 (table 3). To account for genomic inflation, 10 000 permutations were performed and the haplotypes in CFA2 and CFA14 QTL regions remained significant making these regions prime candidates for further genetic studies (table 3). The AE, FG and diameter F traits respectively associated to those two regions explained as much as 13% of the variance of unambiguous CM cases. The haplotype surrounding those two significant QTL may therefore contribute to a significant amount of the variability of CM.

**Table 3:** Raw and permutation *P* values of the haplotypes surrounding all 5 associated QTLs in linear and mixed regression models.

Chr	Associated trait	Position	Regression type	Affected frequency	Unaffected frequency	*P* value*	*P* value after permutations*
2	Diameter F	66803513–67333070	Linear	0.189	0.389	0.0061	0.0421
9	BC	3100030–5716798	Linear	0.070	0.187	0.0318	0.1833
12	Angle 5	11777717–15180310	Mixed	0.235	0.063	0.0106	0.0583
14	AE and FG	51196922–53681140	Mixed	0.085	0.259	0.003	0.0094
24	BC	42012870–44336382	Linear	0.040	0.125	0.0544	0.1293

*Significance threshold set to P value<0.05.

**Figure 4:**
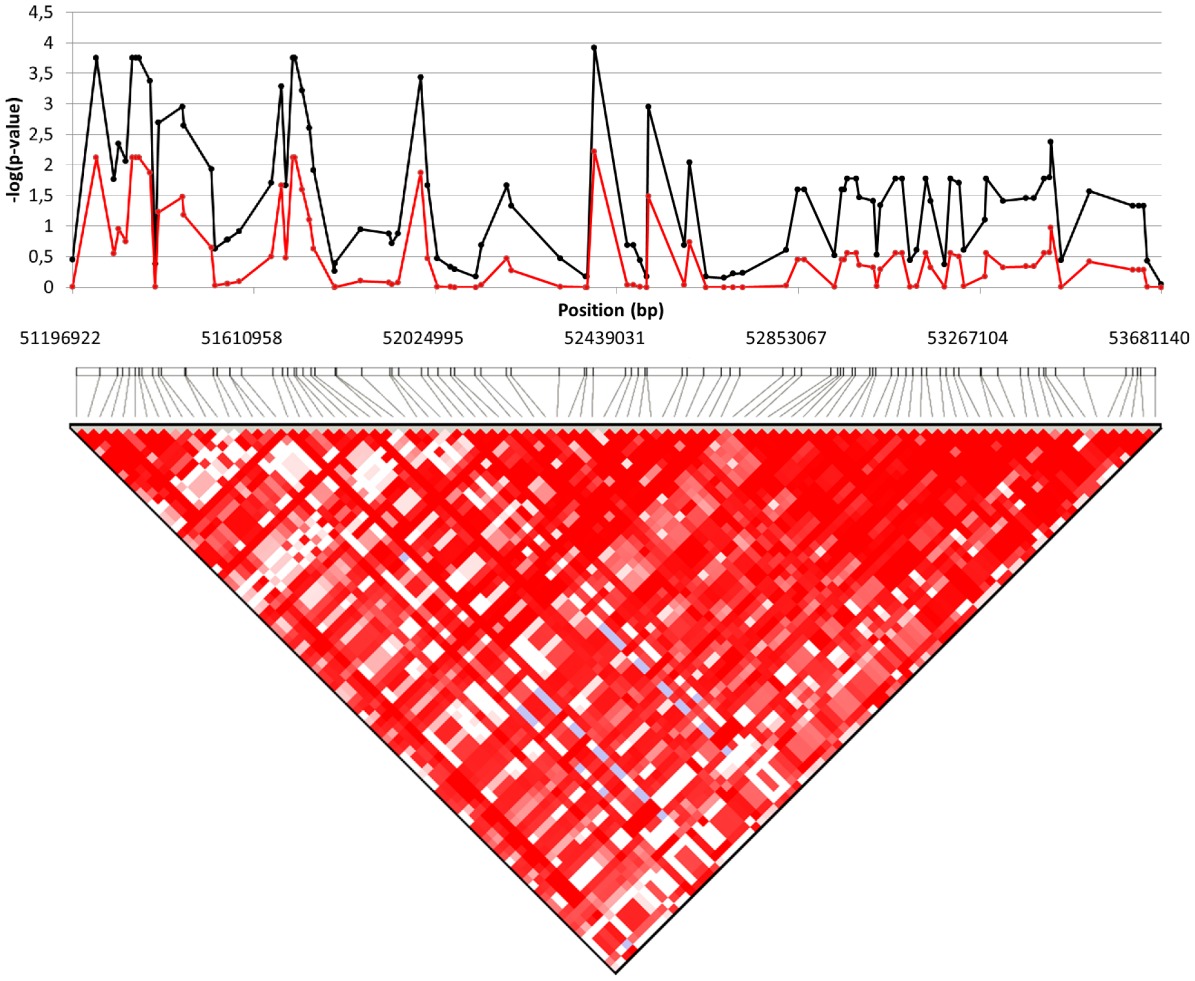
*P* value distribution inside the CFA14 (51196922–53681140 bp) QTL region. *P* value distribution inside the CFA14 QTL region before and after 10 000 permutations. The black line represents the initial CM association score of the SNPs. The red line represents the association score after permutations.

**Figure 5:**
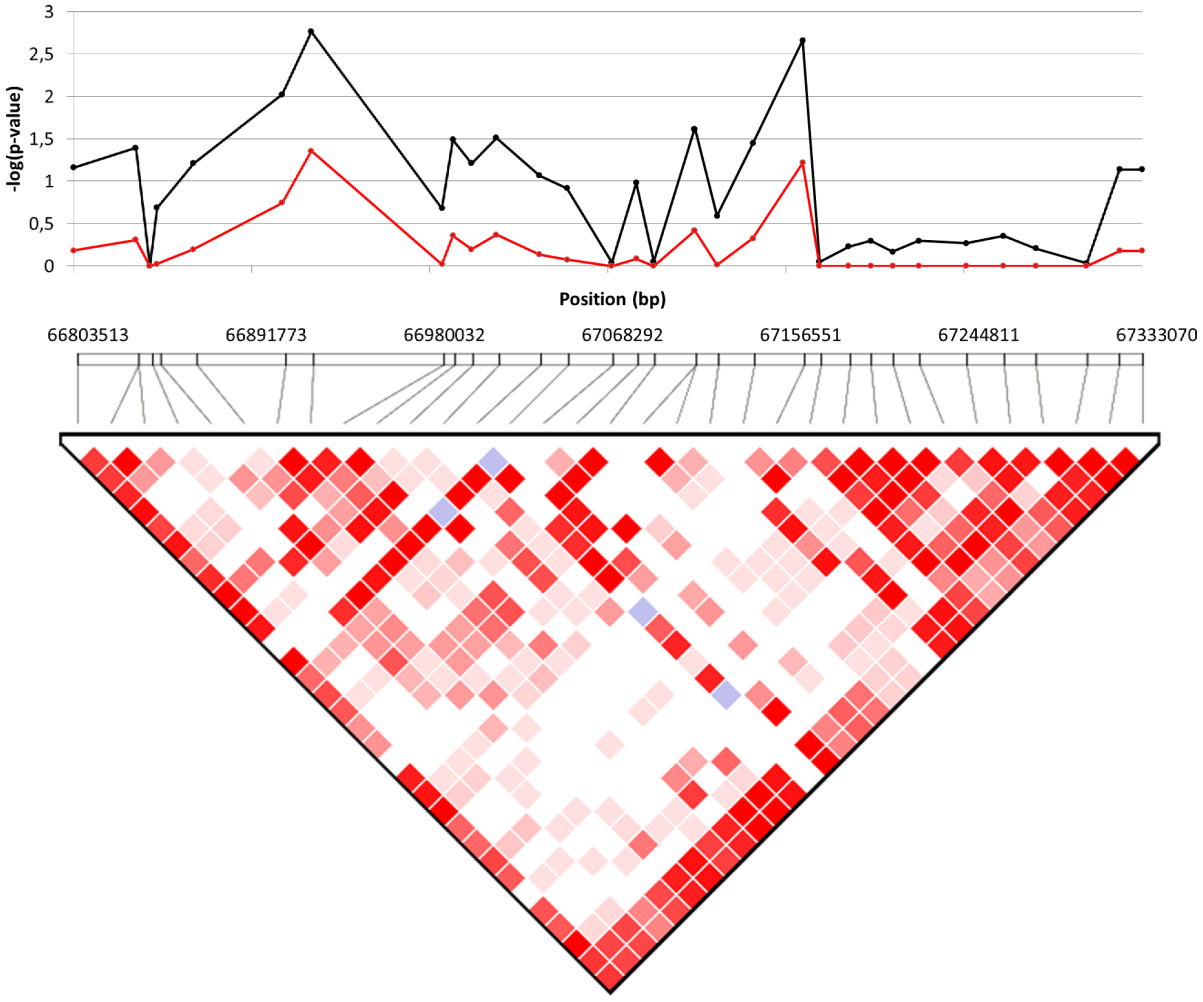
*P* value distribution inside the CFA2 (66803513–67333070 bp) QTL region. *P* value distribution inside the CFA2 QTL region before and after 10 000 permutations. The black line represents the initial CM association score of the SNPs. The red line represents the association score after permutations.

Further investigation of the CFA14 QTL region spanning from 51196922 (rs22371019) to 53681140 bp (rs22291992) (2.48 Mb) resulted in the identification of two significantly associated haplotypes (*P* values of 0.0119 and 0.0094 after 10 000 permutations) (table 4). The 93 SNPs in this region were also tested for association with CM before and after permutations to possibly identify a cluster of strong SNPs that would help pinpoint the associated gene. This resulted in the identification of enrichment in high *P* values in the first half of the haplotype which may contain our candidate gene (figure 4). The CFA2 regions spanning from 66803513 (BICF2G630495282) to 67333070 (rs9236400) (0,53 Mb) and containing 33 SNPs only harboured one significantly associated haplotype (*P* value of 0.0421 after permutations). SNPs in this region were also investigated for clustering of strong *P* values but none was found (table 5 and figure 5).

**Table 4:** Raw and permutation *P* values of the 5 most frequent CM-associated haplotypes in the CFA14 QTL region.

Haplotype	Chromosome	Frequency	Affected frequency	Unaffected frequency	*P* value*	*P* value after permutations*
1	14	0.245	0.179	0.389	0.0038	0.0119
2	14	0.177	0.160	0.056	0.058	0.3057
3	14	0.171	0.170	0.130	0.5079	1.0000
4	14	0.121	0.085	0.259	0.003	0.0094
5	14	0.054	0.066	0	0.0535	0.2921

*Significance threshold set to P value<0.05.

**Table 5:** Raw and permutation *P* values for the 5 most frequent CM-associated haplotypes in the CFA2 QTL region.

Haplotype	Chromosome	Frequency	Affected frequency	Unaffected frequency	*P* value*	*P* value after permutations*
1	2	0.248	0.189	0.389	0.0061	0.0421
2	2	0.124	0.104	0.204	0.0822	0.5590
3	2	0.114	0.189	0.093	0.1135	0.6516
4	2	0.087	0.160	0.037	0.0226	0.1545
5	2	0.087	0.038	0.056	0.6023	1.0000

*Significance threshold set to P value<0.05.

### QTLs suggestive of association

When complex traits are investigated, statistical power is often insufficient to capture all the genetic variations contributing to these traits. This is due to the complex nature of the traits which may be caused by a combination of genes with small effects. Looking at regions that are suggestive of association in the linear model will therefore increase our chance of capturing small effect size genes. To do so, SNPs with *P* values suggestive of association associated to the traits AE, angle 2, angle 5, BC, diameter F and FG were investigated. We identified 30 SNPs suggestive of association in these traits with linear regression models which clustered in 12 QTL genomic regions (Table S1). Haplotype reconstruction around these 12 QTL successfully identified 8 haplotypes that were associated to CM. Correction for genomic inflation using 10 000 permutations left only 5 potential QTL haplotypes associated to CM (Table S2). Those regions need to be further investigated in larger cohorts before concluding on their candidacy.

## Discussion

### MRI skull measurements: important quantitative traits that define the Chiari like malformation

This study shows that multiple quantitative traits can be used in dogs to better describe the CM disease and better capture the genetic variability that underlies it. This suggests that head conformation may play an important role in the increased prevalence of this disease. This idea is also supported by the fact that CM seems to have an increased prevalence in other brachycephalic toy dog breeds while never being reported in non-brachycephalic breeds. While the brachycephalic features of the GB have been previously linked to BMP3 (Bone Morphogenetic Protein 3), we did not see any association for any of the quantitative traits at this locus [13], [20].

Previous morphometric studies conducted in CKCS and GB breeds demonstrate the importance of different morphometric traits in the development of CM. Our main significant locus on CFA2 which has shown association to both diameter F and CM reflects the height of the cranial fossa and is hypothesized to be a compensation for cranial base shortening. While no other loci reached statistical significance in both the QTL analysis and the subsequent haplotype association to CM, CFA14 remains an interesting candidate region for CM. Indeed this region was suggestive of association in the linear model and gave the highest P scores for 2 different traits (length AE and FG) after correction for stratification. This region was also significantly linked to the CM phenotype by the haplotype analysis after 10 000 permutations making it another interesting candidate. Length FG reflects the compensatory increase in the height of the brain caused by the foreshortening of the sphenoid bones in the basicranium which was previously reported in CM affected GB [2]. Length AE may either reflect increased height of the rostral part of the caudal cranial fossa (pars rostralis) or a shift in position of the cerebellum versus the occipital lobes. Interestingly, a compensatory increase in height of the rostro-caudal part of the cranial fossa that could be reflected by the AE and diameter F traits was previously found in both CM affected CKCS and GB breeds [2], [3]. While AE could also represent the position of the occipital lobes relative to the cerebellum, these data suggest that diameter F, length AE and length FG are excellent candidates for QTL studies. The other 3 CM-associated quantitative traits also reflected part of the rostro-caudal and compensatory parietal bone lengthening, but may have been too genetically complex resulting in the absence of significant *P* values.

### QTL regions on CFA2 and CFA14 represent strong candidates for Chiari like malformation in Griffon Bruxellois

Our genome wide QTL study in a cohort of 74 dogs with unambiguous phenotypes based on their CM disease status successfully identified 2 QTL regions on CFA2 and CFA14 that were strongly associated to CM. These two regions which were initially identified as associated to diameter F and both length AE and FG give an insight in the complex genetics of CM in the GB breed. The presence of 1 to 2 haplotypes in these 2 regions that were significantly associated to CM under 10 000 permutations strongly suggests that they harbor genetic variants that are implicated in the development of the disease. Using this two stage approach combining regression and haplotype analysis, we have obtained power to detect potential associated haplotypes that would have been missed using only the initial regression analyses. While only protective haplotypes were identified for both regions, some causative haplotype frequencies could also be observed but our small cohort size and the affected-unaffected bias may have limited statistical power to detect causative haplotypes with smaller effects.

The QTL region on CFA2 spans 0.53 Mb (from 66803513 to 67333070 bp) and contains only the *Sall-1* gene. This gene is a zinc finger transcriptional repressor that may be part of the NuRD histone deacetylase complex (HDAC). Defects in the human orthologue of this gene, *SALL-1*, are a cause of Townes-Brocks syndrome (TBS) (OMIM #107480) as well as branchio-oto-renal syndrome (BOR) (OMIM # #113650) [21], [22]. BOR is characterised by renal and branchial arch anomalies including malformations of the temporal bone and ears [22], [23]. Townes-Brocks syndrome is characterized by renal abnormalities, imperforate anus, thumb malformations with dysplastic ears often associated with sensorineural and/or conductive hearing impairment [21]. In addition ∼10% of patients exhibit neural or behavioral abnormalities and an association of this disease with Chiari type 1 malformation (CMI) in humans has been reported [24], [25]. The link between TBS and CMI represents the canine *Sall-1* gene in the QTL candidate region on CFA2 as an excellent candidate for further genetic and functional studies. The 2.5 Mb region on CFA14 (from 51196922 to 53681140 bp) contains 7 genes, *Elmo1*, *Gpr141*, *Thap5*, *Dnajb9*, *Atp6v1e1*, *Ldha* and *Immp2l*. None of these genes stands out as a strong candidate for CM. A targeted next generation sequencing approach of both QTL regions will be adopted to identify the underlying mutation(s) associated with CM in the GB breed.

While QTL analysis represents a strong tool used to identify associations between multiple traits and diseases, our limited cohort size did not suffice to obtain a strong genetic portrait of this complex disease. While the genetic complexity of this disease has probably been reduced by the high homogeneity of a purebred dog, it remained largely oligogenic and no single major locus could be found. However, our study has successfully identified new QTL associated to CM and provides novel insights into the complex genetics of this disease. Clearly additional genetic studies in larger cohorts and/or novel genomic approaches (for example whole genome or targeted exome sequencing) are needed to further investigate these regions and better define their contribution to CM in the dog.

### CM in the dog: a model for studying Chiari malformation I in humans

CM in the dog is very similar to a condition in humans called Chiari malformation I with a reported frequency of 1 in 1280 [26], [27]. Similarly to CM in dogs, a strong association exists between the size of the skull and the development of the disease in humans [28]–[30]. CMI usually results from a volume discrepancy between the posterior cranial fossa and the neural tissue residing within it resulting in the displacement of the cerebellar tonsil through the foramen magnum. The etiology of CMI is thought to be multifactorial involving genetic factors that remain largely undetermined. Recently, a whole genome linkage study conducted in humans affected with CMI identified multiple associated genomic regions, none of which was syntenic to the CM-associated regions detected in the dog in our study. Results from genetic studies of human CMI should be interpreted cautiously as they are complicated by clinical heterogeneity of the disease and its multifactorial etiology. Additional parallel genetic studies in larger cohorts of human patients and in the dog model are needed to further investigate candidate CM regions identified in both species. It is clear from genetic studies in both humans and dogs that CM or CMI has a complex genetic architecture that would require various complementary approaches to identify the predisposing genetic factors. Nevertheless, the dog model is the only known naturally-occurring animal model for CMI in humans. Hence, gene identification studies in CM in the dog might provide an entry point for identification of novel genes and pathways involved in the pathogenesis of CMI in humans.

## Supporting Information

Table S1 **Frequencies and** ***P*** **values for haplotypes surrounding all QTLs that were suggestive of association in linear regression model.**
(DOCX)

Table S2 **Frequencies and** ***P*** **values of CM-associated haplotypes that were identified in QTLs suggestive of association.**
(DOCX)
