# Supplementary material for: Quantitative Trait Loci (QTL) Study Identifies Novel Genomic Regions Associated to Chiari-Like Malformation in Griffon Bruxellois Dogs
Source: PLoS One. 2014 Apr 16;9(4):e89816. doi: 10.1371/journal.pone.0089816 (PMC3989173; doi:10.1371/journal.pone.0089816)
Supplement: Table S1 — Frequencies and P values for haplotypes surrounding all QTLs that were suggestive of association in linear regression model. (DOCX) [file pone.0089816.s001.docx]

Table S1: Frequencies and *P* values for haplotypes surrounding all QTLs that were suggestive of association in linear regression model

| Chr | Position | Trait | Affected frequency | Unaffected frequency | *P* value |
| --- | --- | --- | --- | --- | --- |
| 2 | 66803513-67333070 | DiamF | 0.189 | 0.389 | 0.0061 |
| 4 | 45961571-46872465 | Ang2 | 0.05 | 0 | 0.115 |
| 6 | 72265391-73666769 | Ang2 | 0 | 0.146 | 9.1361E-5 |
| 7 | 58057157-60298012 | BC | 0.160 | 0 | 0.0033 |
| 9 | 4059047-4522141 | BC | 0.040 | 0 | 0.1601 |
| 13 | 59614046-61400294 | Angle2 | 0 | 0.188 | 8.0322E-6 |
| 14 | 51196922-53681140 | AE and DiamF | 0.085 | 0.259 | 0.0030 |
| 20 | 21154534-22752895 | BC | 0.131 | 0.333 | 0.0039 |
| 22 | 17164695-18492974 | BC | 0.071 | 0 | 0.059 |
| 24 | 41874084-44327660 | BC | 0.070 | 0.167 | 0.0682 |
| 28 | 21565968-23102979 | BC | 0.020 | 0.250 | 7.6154E-6 |
| 33 | 8067399-10706890 | Angle5 | 0.220 | 0.417 | 0.013 |

*Significance threshold set to P value < 0.05
