# Supplementary material for: Quantitative Trait Loci (QTL) Study Identifies Novel Genomic Regions Associated to Chiari-Like Malformation in Griffon Bruxellois Dogs
Source: PLoS One. 2014 Apr 16;9(4):e89816. doi: 10.1371/journal.pone.0089816 (PMC3989173; doi:10.1371/journal.pone.0089816)
Supplement: Table S2 — Frequencies and P values of CM-associated haplotypes that were identified in QTLs suggestive of association. (DOCX) [file pone.0089816.s002.docx]

| Chr | Position | Trait | Affected frequency | Unaffected frequency | *P* value | *P* value after 10000 permutations |
| --- | --- | --- | --- | --- | --- | --- |
| 6 | 72265391-73666769 | Angle 2 | 0 | 0.146 | 9.1361E-5 | 0,0005 |
| 7 | 58057157-60298012 | BC | 0.160 | 0 | 0.0033 | 0.0258 |
| 13 | 59614046-61400294 | Angle2 | 0 | 0.188 | 8.0322E-6 | 8.03E-6 |
| 20 | 21154534-22752895 | BC | 0.131 | 0.333 | 0.0039 | 0.0423 |
| 28 | 21565968-23102979 | BC | 0.020 | 0.250 | 7.6154E-6 | 7.6154E-6 |

Table S2: Frequencies and *P* values of CM-associated haplotypes that were identified in QTLs suggestive of association

*Significance threshold set to P value < 0.05
